# Supplementary material for: Melanoma Cell State-Specific Responses to TNFα
Source: Biomedicines. 2021 May 26;9(6):605. doi: 10.3390/biomedicines9060605 (PMC8230114; doi:10.3390/biomedicines9060605)
Supplement: Supplementary file 1 [file biomedicines-09-00605-s001.zip › biomedicines-1232290-supplementary.pdf]

Supplementary Figure 1

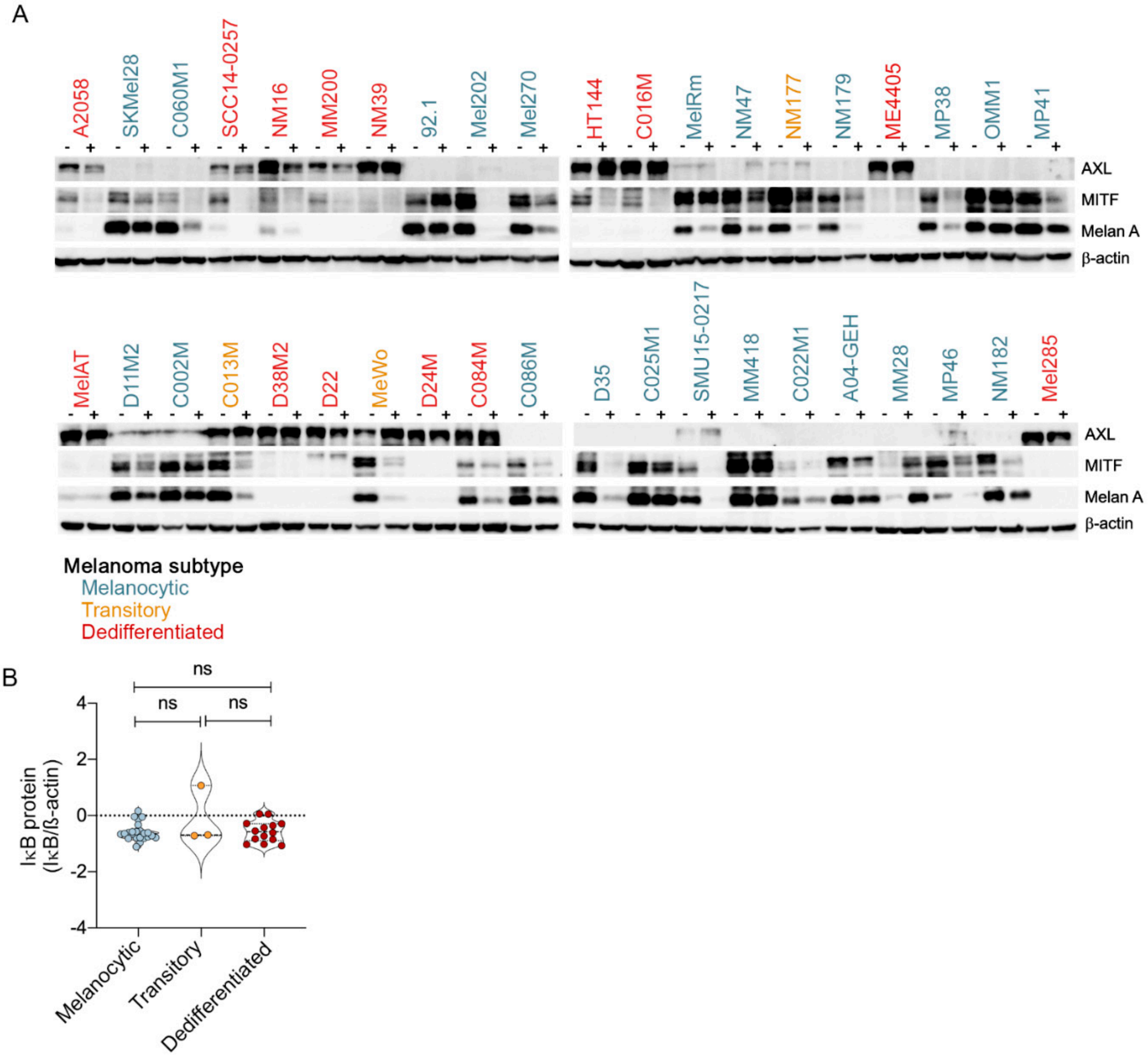

Supplementary Figure S1. Expression of melanoma subtype signatures in response to TNFα. (**A**) Western blots of melanocytic (blue), transitory (orange) and dedifferentiated (red) melanoma cell lysates showing AXL, MITF and Melan A accumulation, 72 h after treatment with BSA control (-) or TNFα (+). (**B**) TNFα-induced IκB expression (72 h after TNFα treatment; values normalized to ß-actin and converted to z-scores) in melanocytic (blue), transitory (orange) and neural crest-like dedifferentiated (red) melanoma cell lines. Each dot represents the average of three independent experiments for each cell line. Comparisons between groups were determined by one-way ANOVA and Tukey's multiple comparisons test.

Supplementary Figure 2

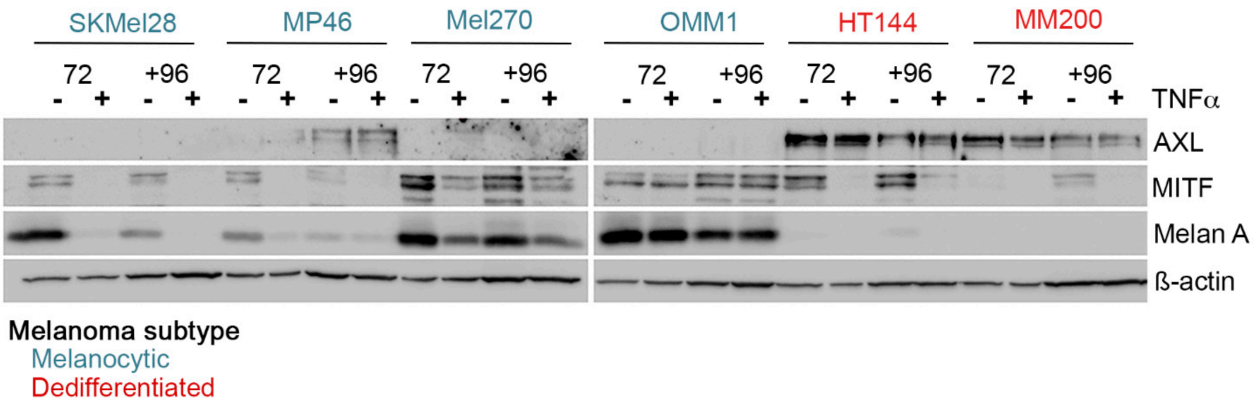

Supplementary Figure S2. TNFα-induced dedifferentiation is reversible. Western blots of melanocytic (SKMel28, MP46, MEL270, OMM1) and dedifferentiated (HT144, MM200) melanoma cell lysates showing AXL, MITF and Melan A expression 72 h (72) after treatment with BSA control (-) or TNFα (+). TNFα was either removed (-) or added (+) for another 96 h (+96).

Supplementary Figure 3

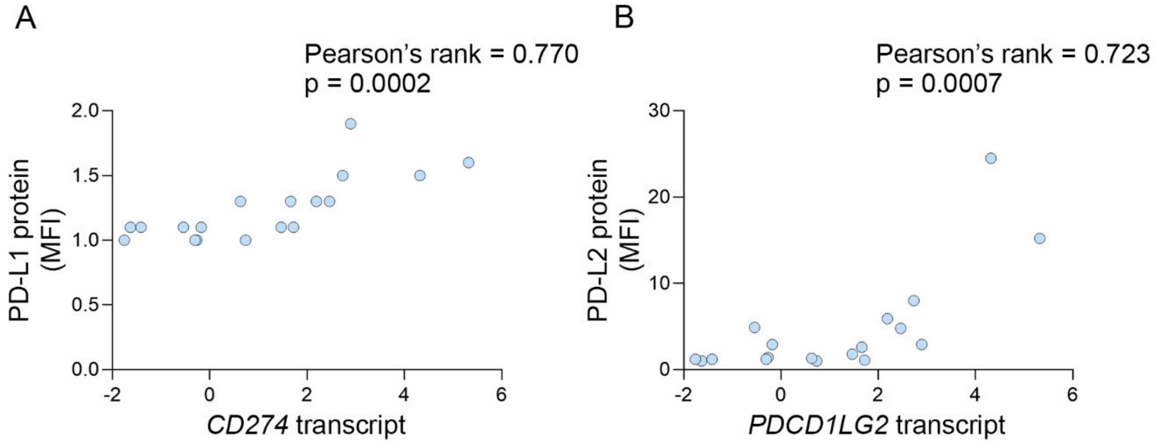

Supplementary Figure S3. PD-L1 and PD-L2 protein and transcript expression. Scatterplots showing correlation of A) PD-L1 protein with the CD274 transcript and B) PD-L2 protein with the PDCD1LG2 transcript at baseline and post TNFα treatment in nine melanoma cell lines with matched transcriptome data. Correlation calculated using Pearson's rank correlation coefficient, $p < 0.05$.
